# Supplementary material for: Cardiac endothelial cells maintain open chromatin and expression of cardiomyocyte myofibrillar genes
Source: eLife. 2020 Dec 14;9:e55730. doi: 10.7554/eLife.55730 (PMC7758065; doi:10.7554/eLife.55730)
Supplement: Transparent reporting form [file elife-55730-transrepform.docx]

***eLife’s* transparent reporting form**

We encourage authors to provide detailed information *within their submission* to facilitate the interpretation and replication of experiments. Authors can upload supporting documentation to indicate the use of appropriate reporting guidelines for health-related research (see [EQUATOR Network](about:blank)), life science research (see the BioSharing Information Resource), or the [ARRIVE guidelines](about:blank) for reporting work involving animal research. Where applicable, authors should refer to any relevant reporting standards documents in this form.

If you have any questions, please consult our Journal Policies and/or contact us: [editorial@elifesciences.org](about:blank).

**Sample-size estimation**

- You should state whether an appropriate sample size was computed when the study was being designed
- You should state the statistical method of sample size computation and any required assumptions
- If no explicit power analysis was used, you should describe how you decided what sample (replicate) size (number) to use

Please outline where this information can be found within the submission (e.g., sections or figure legends), or explain why this information doesn’t apply to your submission:

No explicit power analysis was used. For analysis of deposited data, sample size was limited by the replicates provided. For ATACSeq, two replicates were used, as according to the ENCODE current stadards for ATAC-Seq ([https://www.encodeproject.org/atac-seq/](about:blank))

**Replicates**

- You should report how often each experiment was performed
- You should include a definition of biological versus technical replication
- The data obtained should be provided and sufficient information should be provided to indicate the number of independent biological and/or technical replicates
- If you encountered any outliers, you should describe how these were handled
- Criteria for exclusion/inclusion of data should be clearly stated
- High-throughput sequence data should be uploaded before submission, with a private link for reviewers provided (these are available from both GEO and ArrayExpress)

Please outline where this information can be found within the submission (e.g., sections or figure legends), or explain why this information doesn’t apply to your submission:

Two biological replicates (different mice processed on different days) were used for ATAC each with 25-50 million unique paired ends reads. This data can be found under the ‘ATACSeq’ paragraph of the methods section. Sequencing data has been deposited in GEO under the accession number GSE144839. Reviewers can use the token wfgteywmbbsvzah to access data while it remains under private status.

**Statistical reporting**

- Statistical analysis methods should be described and justified
- Raw data should be presented in figures whenever informative to do so (typically when N per group is less than 10)
- For each experiment, you should identify the statistical tests used, exact values of N, definitions of center, methods of multiple test correction, and dispersion and precision measures (e.g., mean, median, SD, SEM, confidence intervals; and, for the major substantive results, a measure of effect size (e.g., Pearson's r, Cohen's d)
- Report exact p-values wherever possible alongside the summary statistics and 95% confidence intervals. These should be reported for all key questions and not only when the p-value is less than 0.05.

Please outline where this information can be found within the submission (e.g., sections or figure legends), or explain why this information doesn’t apply to your submission:

Statistics used are reported in the Methods section. For all graphs error bars shown are SEM. For statistics of sequencing data, DESEQ2 was used in the DiffBind R package to identify significant changes. False discovery rate (FDR) corrected p value < 0.05 was used to determine significance. Unless otherwise indicated, n=3 biological replicates were used for non-sequencing data. For GO and HOMER analysis, FDR-corrected p-values are shown (indicated in figures).

(For large datasets, or papers with a very large number of statistical tests, you may upload a single table file with tests, Ns, etc., with reference to sections in the manuscript.)

**Group allocation**

- Indicate how samples were allocated into experimental groups (in the case of clinical studies, please specify allocation to treatment method); if randomization was used, please also state if restricted randomization was applied
- Indicate if masking was used during group allocation, data collection and/or data analysis

Please outline where this information can be found within the submission (e.g., sections or figure legends), or explain why this information doesn’t apply to your submission:

Specific masking of data was not used. Sample comparison of sequencing data was done via unbiased statistics.

**Additional data files (“source data”)**

- We encourage you to upload relevant additional data files, such as numerical data that are represented as a graph in a figure, or as a summary table
- Where provided, these should be in the most useful format, and they can be uploaded as “Source data” files linked to a main figure or table
- Include model definition files including the full list of parameters used
- Include code used for data analysis (e.g., R, MatLab)
- Avoid stating that data files are “available upon request”

Please indicate the figures or tables for which source data files have been provided:

Sequencing data has been deposited in GEO under the accession number GSE144839. Reviewers can use the token wfgteywmbbsvzah to access data while it remains under private status.
